# Prevalence and genotype diversity of *Campylobacter jejuni* in hunted reared pheasants (*Phasianus colchicus*) in Finland

**DOI:** 10.1186/s13028-023-00698-7

**Published:** 2023-08-01

**Authors:** Rauni Kivistö, Mikaela Sauvala, Maria Fredriksson-Ahomaa, Johanna Björkroth

**Affiliations:** Department of Food Hygiene and Environmental Health, Faculty of Veterinary Medicine, University of Helsinki, Agnes Sjöbergin katu 2, Helsinki, FI-00790 Finland

**Keywords:** Campylobacteriosis, *C. jejuni*, Game bird, Hunting, One Health, Pathogen, Poultry, Wild bird, Zoonosis

## Abstract

*Campylobacter* spp., especially *C. jejuni*, is the most common zoonotic pathogen in humans worldwide. In Nordic countries, the prevalence of *C. jejuni* in broilers, which is an important reservoir of human infections, is generally low. Thus, other sources of domestically acquired infections besides chicken meat need to be considered. Game birds are known to carry a variety of zoonotic agents, including *Campylobacter*. The aim of this study was to investigate *Campylobacter* spp. carriage in a flock of reared pheasants at hunting in two successive samplings to better understand the dynamics of *Campylobacter* infections in pheasants. Overall, 72% of the intestinal samples were positive for *Campylobacter* spp. by direct culture on mCCDA. *C. jejuni* was the only species identified. The isolates were genotyped using whole genome sequencing (WGS), multilocus sequence typing (MLST), and *ad hoc* whole genome MLST (wgMLST). Two distinct *C. jejuni* clones were identified among the 18 isolates studied, representing MLST sequence types (STs) ST-45 and ST-699. The ST-45 isolates were closely related to previous human clinical isolates using core genome MLST (cgMLST). In contrast, the ST-699 isolates forming the dominant clone in the latter sampling were quite distinct from previously described cgMLST profiles from different hosts and sources worldwide. In conclusion, the intestine of reared pheasants is commonly colonized by *C. jejuni* and may carry genotypes relevant to infections in livestock and humans. Hygienic measures are needed to limit the spread of infection in reared flocks. Especially farmers and hunters having direct contact with pheasant offal need to be aware of the associated zoonosis risk to protect themselves and their working dogs alike. Biosecurity measures to improve the safety and reduce the zoonosis risk associated with pheasant farming should be further investigated.

**Supplementary Information:**

The online version contains supplementary material available at 10.1186/s13028-023-00698-7.

## Findings

*Campylobacter jejuni* is the most common zoonotic pathogen worldwide, including Finland and other parts of Scandinavia [1], with broiler meat and milk being the most frequent causes of foodborne outbreaks. Compared to EU average, *Campylobacter* spp. prevalence in Finnish broiler flocks is low, and the consumption of fresh chicken meat has been estimated to account only for 18–24% of domestically acquired sporadic campylobacteriosis cases, based on whole genome multilocus sequence typing (wgMLST) [2, 3]. Thus, other sources and transmission routes are likely to exist. A recent study from the Netherlands attributed campylobacteriosis cases based on high-resolution genomic data to chickens or turkeys (48.2%), dogs or cats (18.0%), cattle (12.1%), and surface water (8.5%) [4]. Further case exposure analysis revealed open water swimming, contact with dog faeces, and consumption of non-chicken or turkey avian meat, such as game bird meat from ducks, geese, quails, and pheasants, as other environmental risk factors. Previously a cluster of campylobacteriosis cases has been linked to occupational exposure to pheasants [5]. The cohort study noted high attack rates among workers with direct contact to pheasant faeces and first-time workers highlighting the possible role of pheasants in *Campylobacter* infections.

In Finland, due to harsh winter conditions, the natural pheasant (*Phasianus colchicus*) population is restricted to southern parts of the country thriving only in the vicinity of bird sanctuaries and other feeding places. For hunting purposes pheasants are also hatched and grown on local farms. Pheasant meat is also sold to restaurants. Previous studies reported 9% prevalence of *C. jejuni* by culture [6] and 21.8% by polymerase chain reaction (PCR) [7] among reared wild hunted pheasants in Finland. Other countries report *Campylobacter* spp. prevalence ranging from 0 to 73% [7–12]. However, only two previous studies genotyped the isolates to better understand their association with human infections [6, 11]. Sequence type ST-19, previously recovered also from humans, poultry, cattle, and sheep, being the most common *C. jejuni* MLST sequence type identified. The Finnish ST-19 clone was resistant to ciprofloxacin, although resistance in Finnish poultry production is generally low [13] in line with the limited to non-existent use of antibiotics.

Our aim was to describe the *Campylobacter* population in a flock of reared pheasants sampled twice at hunting, one month apart, using whole genome sequencing (WGS), MLST, and core genome MLST (cgMLST). We wanted to find out whether the flock was colonized by several different clones of *Campylobacter*, how persistent the colonization was, and whether the ciprofloxacin-resistant ST-19 clone continued to predominate in Finnish pheasants.

No animals were killed for the purpose of this study. From a flock of 500 pheasants reared for hunting purposes on a game bird farm in South-West Finland, 20 to 30 pheasants were released from their pen in the morning of each hunt. The birds were hunted with the help of pointing dogs and shot by licensed hunters first in October (n = 12) and later in November 2018 (n = 13) at the age of approximately 21 and 26 weeks, respectively. Predatory animals (foxes, mustelids, goose, hawks etc.) are also common in the area, and thus the pheasants escaping the hunt will not survive to form a wild population.

After shooting, the birds were immediately eviscerated at site by a veterinarian working carefully (wearing gloves) not to cross-contaminate the samples. No punctures in the intestines were observed and the intestines were individually disposed into plastic bags for transport refrigerated to the laboratory. The samples were cultivated directly on mCCDA plates incubated under microaerobic conditions at 41.5 ˚C for 48 h. One typical colony per sample was confirmed as *Campylobacter* spp. by the lack of aerobic growth on blood agar at 25 ˚C, Gram-stain, genus- and species-specific PCR [14]. Pure cultures were grown on nutrient blood agar at 37 ˚C under microaerobic conditions. Genomic DNA was extracted using PureLink™ Genomic DNA Mini Kit and stored at -20 ˚C prior to PCR and sequencing.

WGS was performed at the Institute for Molecular Medicine Finland (FIMM) using Illumina MiSeq. Raw sequence data were assembled using INNUca pipeline [15] (https://github.com/B-UMMI/INNUca). Comparisons between the isolates were performed using ad hoc wgMLST [16]. Minimum Spanning Trees representing pairwise allele distances were visualized using GrapeTree [17]. Antimicrobial resistance determinants were screened for using ResFinder [18]. Finally, *Campylobacter* isolates sequenced in this study were compared against the PubMLST database [19] (last accessed 4th April 2023) using the *C. jejuni/C. coli* cgMLST v1.0 scheme [20]. For a more detailed description of the materials and methods, see Additional file 1.

**Fig. 1:**
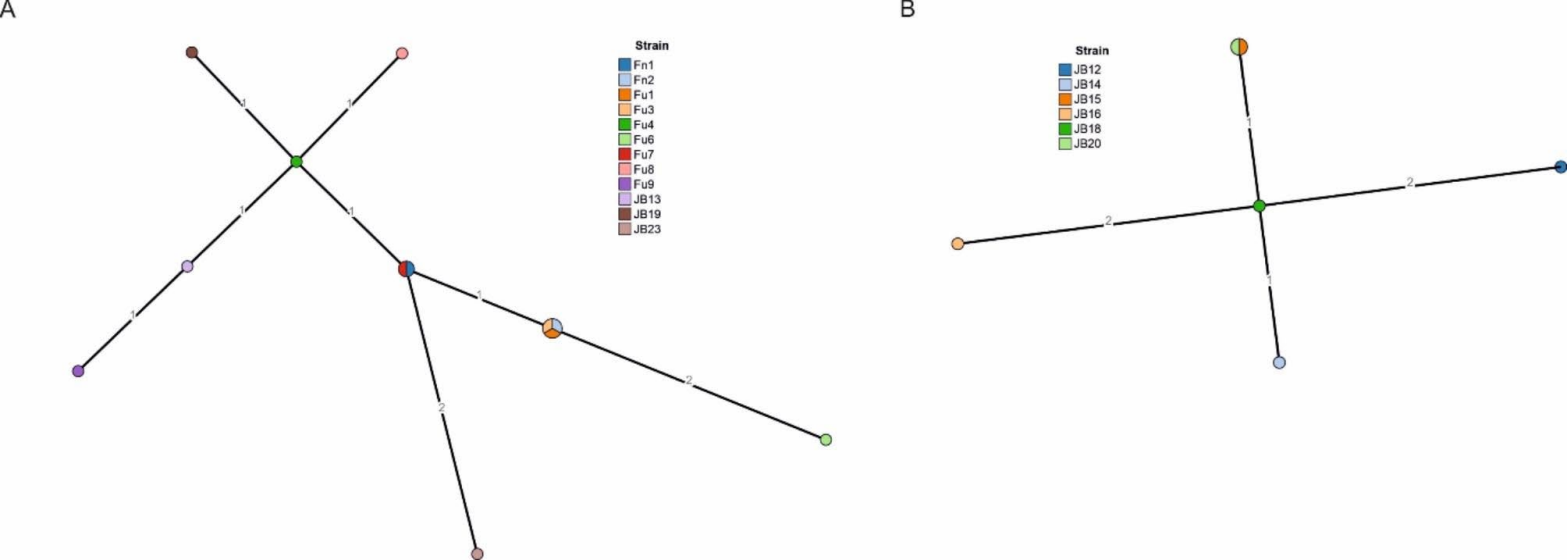
Minimum spanning tree (MST) results of *ad hoc* wgMLST analysis of the pheasant *C. jejuni* isolates. Panels **A** and **B** show MSTs of ST-45 (1709 shared loci) and ST-699 (1733 shared loci), respectively. The numbers on the branches indicate pairwise allelic distances between each node of the tree

*Campylobacter* spp. were isolated from 75% (95% CI: 43–95%) and 69% (95% CI: 39–91%) of samples in the first and second batch, respectively (Table 1). *C. jejuni* was the only species identified. In October ST-45 was the only sequence type detected. In November both ST-699 (67%; 95% CI: 30–93%) and ST-45 were identified among the positive samples. In wgMLST analysis the strains formed two separate highly clonal clusters (Fig. 1). No antimicrobial resistance determinants, other than the blaOXA-184 Class D beta-lactamase, were found. Query of the PubMLST database with Fu7 (ST-45) draft genome revealed a single linkage Cjc_cgc_10 cluster with isolates from humans (n = 9; years 2012–2018) and chicken (n = 8; 2014 and 2016) from the UK, and an urban rat from Finland (2021). JB18 (ST-699) was un-associated with previously deposited isolates.

**Table 1:** Multilocus sequence typing results of *C. jejuni* isolates collected from a flock of farmed hunted pheasants from two successive samplings

Sample ID	Date	*Campylobacter* status (species identified)	MLST	Sample ID	Date	*Campylobacter* status (species identified)	MLST
Fn1	7.10.2018	pos. (*C. jejuni*)	ST-45	JB11	11.11.2018	neg.	
Fn2	7.10.2018	pos. (*C. jejuni*)	ST-45	JB12	11.11.2018	pos. (*C. jejuni*)	ST-699
Fu1	7.10.2018	pos. (*C. jejuni*)	ST-45	JB13	11.11.2018	pos. (*C. jejuni*)	ST-45
Fu2	7.10.2018	neg.		JB14	11.11.2018	pos. (*C. jejuni*)	ST-699
Fu3	7.10.2018	pos. (*C. jejuni*)	ST-45	JB15	11.11.2018	pos. (*C. jejuni*)	ST-699
Fu4	7.10.2018	pos. (*C. jejuni*)	ST-45	JB16	11.11.2018	pos. (*C. jejuni*)	ST-699
Fu5	7.10.2018	neg.		JB17	11.11.2018	neg.	
Fu6	7.10.2018	pos. (*C. jejuni*)	ST-45	JB18	11.11.2018	pos. (*C. jejuni*)	ST-699
Fu7	7.10.2018	pos. (*C. jejuni*)	ST-45	JB19	11.11.2018	pos. (*C. jejuni*)	ST-45
Fu8	7.10.2018	pos. (*C. jejuni*)	ST-45	JB20	11.11.2018	pos. (*C. jejuni*)	ST-699
Fu9	7.10.2018	pos. (*C. jejuni*)	ST-45	JB21	11.11.2018	neg.	
Fu10	7.10.2018	neg.		JB22	11.11.2018	neg.	
				JB23	11.11.2018	pos. (*C. jejuni*)	ST-45

We found a relatively high prevalence (72%) of *Campylobacter* spp. in reared game pheasants. Previously, 27% (4/15) of pheasants were found to be positive by culture methods in Russia [12], 44% (4/9) in north-western Italy [10], 43% (104/240) in southern Italy [9], and 26% (14/52) in Germany [8]. In Scotland, 37% (105/287) of the samples were found to be positive, ranging from 0 to 73% prevalence between different estates [11]. The age of the birds might partly explain the high prevalence observed in our study since previously the prevalence of *Campylobacter* spp. has also been shown to be higher in adult (> 1 month, 83.3%) compared to younger pheasants (< 1 month, 3.3%) [9].

The pheasants were grown and fed exclusively in their pen and released to the farm surroundings in the morning of the hunt. By contrast, in our previous study [6], pheasants were released already as 4-week-olds with continued supplementary feeding. The latter birds raised in less crowded conditions showed a significantly lower prevalence of *C. jejuni* (9%) [6] than the birds (72%) reared more intensively in this study. These results are in line with the previous finding that pheasants grown in intensive production up till the age of 5–8 months were more likely to carry *Campylobacter* spp. at the time of hunt than wild pheasants released from the reproductive farm at the age of 8 weeks (70.2% vs. 27.5%) [21], suggesting that early release would be preferable. However, it is important to note that effective predator control is important for the survival of young pheasants in the wild in Finland.

In southern Italy *C. coli* was the dominant *Campylobacter* species present in all positive samples, whereas *C. jejuni* was present only in 13.5% of the samples [9]. Also in Scotland, *C. coli* was more prevalent (62.6%) than *C. jejuni* (37.4% of positive samples), and the risk for *C. coli* infection from pheasants was considered to be much higher [11]. In contrast, only *C. jejuni* was isolated from Finnish pheasants in this and our previous study [6]. Further studies are needed to explain this finding.

*C. jejuni* ST-19, the previously most predominant genotype in pheasants [6, 11], was not identified in this study. However, another multi-host sequence type, ST-45, was found. ST-45 has previously been isolated from human cases of gastroenteritis and systemic disease as well as chicken, cattle, and sheep, but also e.g. environmental waters, ducks, dogs, turkeys, and wild birds [19]. In Finland ST-45 has been the most common ST in chicken slaughter batches since 2004, prevalence ranging from 18.6 to 46.6% [22]. In humans and natural waters, ST-45 has accounted for 21–31% [23, 24] and 30–39% [2, 25] of the isolates, respectively. Further analysis revealed that our ST-45 clone was clearly distinct from those previously described from various sources, however, most closely related to human clinical and chicken isolates from the UK, with WGS in their active surveillance system for years. Since the ST-45 clone persisted in our pheasant flock for at least a month, further studies are needed to reveal if pheasants overwintering in Finland may present an important reservoir for pathogenic *C. jejuni*.

ST-699 has been reported from humans, chicken, goose, wild birds, and environmental waters worldwide, however infrequently [19]. In Finland, ST-699 has only been identified from natural surface waters [25]. cgMLST analysis showed no close hits for our ST-699 clone, yet interestingly, this new *C. jejuni* clone outnumbered the ST-45 clone in November and no other genotypes were observed. This could possibly be explained by the main migration season of wild birds occurring between samplings. However, we cannot completely rule out the possibility of co-colonization of the pheasant with even more clones or species since only one pure culture from each intestine was studied.

In conclusion, pheasants in Finland are commonly colonized by *C. jejuni* and hygienic measures are needed to limit the spread of infection. Farmers, hunters, and veterinarians having direct contact with pheasant offal need to be aware of the associated zoonosis risk to protect themselves and others.

## Electronic supplementary material

Below is the link to the electronic supplementary material.

Additional file 1. Detailed description of materials and methods used in the study.

## Data Availability

The raw sequence data related to the current study have been deposited in the NCBI database under BioProject PRJNA430314 and BioSample accession numbers SAMN18746114 to SAMN18746131.

## Declarations

### Prior publication

Data have not been published previously.
