# Supplementary material for: Prevalence and genotype diversity of Campylobacter jejuni in hunted reared pheasants (Phasianus colchicus) in Finland
Source: Acta Vet Scand. 2023 Aug 1;65:36. doi: 10.1186/s13028-023-00698-7 (PMC10394823; doi:10.1186/s13028-023-00698-7)
Supplement: Supplementary file 1 — Additional file 1. Detailed description of materials and methods used in the study. [file 13028_2023_698_MOESM1_ESM.docx]

**Additional file 1.** Detailed description of materials and methods used in the study.

**Samples**

No animals were killed for the purpose of this study. Pheasants (N = 25) shot by licensed hunters were included in the study. The pheasants were hunted by small hunting parties, each consisting of three persons and a pointing dog to locate the birds for the shooters and to retrieve the birds after they were shot. Birds were hunted from the surroundings (wild forest, field edges, meadows) of a game bird farm (South-West Finland) on two occasions, first in October (n=12) and then in November (n=13) 2018. The farm had purchased 500 chicks from another farm specialized in hatching and producing pheasant chicks and reared them to adulthood. The chicks were born at the end of May, and thus, the age of the birds was approximately 21 and 26 weeks when shot in October and November, respectively. Their feed consisted of commercial feed and grain produced on this farm specialized in organic farming. Part of the pheasants (20-30 birds) were released from the pen to the farm surroundings each morning of the hunt, and thus the birds had very limited contact with wildlife of the area before being shot. No natural population of wild pheasants lived in the area due to the presence of predatory animals (foxes, mustelids, goose, hawks etc.). After shooting, the birds were immediately eviscerated at site by a veterinarian working carefully (wearing gloves) not to cross-contaminate the samples. No punctures in the intestines were observed and the intestines were individually disposed into plastic bags for transport to the laboratory. Due to the cold weather (6-8°C), refrigeration started immediately, and cold temperatures were maintained during transport and prior to all analyses.

**Bacterial isolates**

The intestinal samples were cultivated on mCCDA plates (Oxoid Ltd., Hampshire, UK) by direct culture after the intestinal contents had been homogenized using a cotton swab dipped in Nutrient Broth No. 2 (Thermo Fisher Scientific Inc., Waltham, MA, USA). The plates were incubated under microaerobic conditions (5% O_2_, 10% CO_2_, ≤ 10% H_2_, balanced with N_2_; Anoxomat System, Mart Microbiology, the Netherlands) at 41.5˚C for 48 h. One typical colony per sample was confirmed as *Campylobacter* spp. by the lack of aerobic growth on blood agar at 25˚C, Gram-stain, genus- and species-specific PCR [1]. Pure cultures were grown on nutrient blood agar plates supplemented with bovine blood (Labema Oy, Helsinki, Finland) at 37˚C under microaerobic conditions.

Genomic DNA was extracted using the PureLink™ Genomic DNA Mini Kit (Invitrogen, Waltham, MA, USA) according to the manufacturer’s instructions. The purity of the DNA was tested using NanoDrop (Thermo Fisher Scientific), and quantitated using the Qubit fluorometer (Thermo Fisher Scientific) with the Qubit dsDNA Broad Range Kit (Thermo Fisher Scientific). DNA was stored at -20˚C prior to PCR and sequencing.

**Whole genome sequencing and genome assembly**

Whole genome sequencing (WGS) was performed on all *C. jejuni* isolates (n=18) at the Institute for Molecular Medicine Finland (FIMM) using Illumina MiSeq Technology (Illumina, San Diego, CA, USA) with a 300-bp paired-end protocol according to the manufacturer’s instructions, aiming at > 100x coverage. The sequencing libraries were prepared using the Nextera Flex kit (Illumina).

Raw sequence data were assembled into contigs using the INNUca pipeline v4.2.1 [2] (<https://github.com/B-UMMI/INNUca>). In short, INNUca checks read quality using FastQC (<https://www.bioinformatics.babraham.ac.uk/projects/fastqc/>) and trims the reads using Trimmomatic [3]. Then, de novo draft genome assembly is performed using SPAdes 3.11 [4] (<http://cab.spbu.ru/software/spades/>). Pilon [5] (<https://github.com/broadinstitute/pilon>) is used to improve the draft genome assemblies. A seven-gene MLST sequence type (ST) [6] is auto-assigned with the mlst v2.4 software [7] (<https://github.com/tseemann/mlst>). Finally, the depth of coverage and the lack of contamination are estimated using the ReMatCh software (<https://github.com/B-UMMI/ReMatCh>).

**Multilocus sequence typing (MLST)**

Further comparison between the isolates was performed with a gene-by-gene approach using the chewBBACA suite [8]. The INNUENDO *C. jejuni* wgMLST schema consisting of 2794 loci [9] (<https://zenodo.org/record/1322564#.YHVvO-gzY2w>) was used to define the *ad hoc* wgMLST profiles. Only loci present in all the genomes of a particular set of isolates were included in the final *ad hoc* wgMLST profile. Minimum Spanning Trees (MST) representing pairwise allele distances (i.e. loci differences) were calculated and visualized using GrapeTree [10].

Representative draft genomes (Fu7 and JB18) were uploaded to the PubMLST database [11] (last accessed 4^th^ April 2023) to determine their *C. jejuni/C. coli* cgMLST v1.0 profiles [12] and to determine their single linkage clusters to identify other similar strains from the public isolate database using BIGSdb [11]. Single linkage clusters (Cjc_cgc_10), where each member has less than 10 allelic differences to at least one other member of the group were considered. Missing loci were ignored in the comparisons.

**Sequence deposition**

The raw sequence data were deposited in the NCBI database under BioProject PRJNA430314 and BioSample accession numbers SAMN18746114 to SAMN18746131.

**Antimicrobial resistance**

Antimicrobial resistance determinants were screened from the draft genome assemblies with ResFinder 3.2 (Zankari et al., 2012) using default settings.

**References**

1. Denis M, Soumet C, Rivoal K, Ermel G, Blivet D, Salvat G, et al. Development of a m-PCR assay for simultaneous identification of Campylobacter jejuni and C. coli. Letters in applied microbiology. 1999;29(6):406-10.

2. Machado MP, Halkilahti J, Jaakkonen A, Silva DN, Mendes I, Nalbantoglu Y, et al.: INNUca. In.: GitHub; 2019.

3. Bolger AM, Lohse M, Usadel B. Trimmomatic: a flexible trimmer for Illumina sequence data. Bioinformatics. 2014;30(15):2114-20; doi: <https://doi.org/10.1093/bioinformatics/btu170>.

4. Bankevich A, Nurk S, Antipov D, Gurevich AA, Dvorkin M, Kulikov AS, et al. SPAdes: a new genome assembly algorithm and its applications to single-cell sequencing. J Comput Biol. 2012;19(5):455-77; doi: <https://doi.org/10.1089/cmb.2012.0021>.

5. Walker BJ, Abeel T, Shea T, Priest M, Abouelliel A, Sakthikumar S, et al. Pilon: an integrated tool for comprehensive microbial variant detection and genome assembly improvement. PLoS One. 2014;9(11):e112963; doi: <https://doi.org/10.1371/journal.pone.0112963>.

6. Dingle KE, Colles FM, Wareing DR, Ure R, Fox AJ, Bolton FE, et al. Multilocus sequence typing system for *Campylobacter jejuni*. J Clin Microbiol. 2001;39(1):14-23; doi: <https://doi.org/10.1128/JCM.39.1.14-23.2001>.

7. Seemann T: mlst. In.: GitHub; 2016.

8. Silva M, Machado MP, Silva DN, Rossi M, Moran-Gilad J, Santos S, et al. chewBBACA: A complete suite for gene-by-gene schema creation and strain identification. Microb Genom. 2018; doi: <https://doi.org/10.1099/mgen.0.000166>.

9. Rossi M, Silva MSD, Ribeiro-Gonçalves BF, Silva DN, Machado MP, Oleastro M, et al.: INNUENDO whole genome and core genome MLST schemas and datasets for *Campylobacter jejuni*. In.; 2018.

10. Zhou Z, Alikhan NF, Sergeant MJ, Luhmann N, Vaz C, Francisco AP, et al. GrapeTree: visualization of core genomic relationships among 100,000 bacterial pathogens. Genome Res. 2018;28(9):1395-404; doi: <https://doi.org/10.1101/gr.232397.117>.

11. Jolley KA, Bray JE, Maiden MCJ. Open-access bacterial population genomics: BIGSdb software, the PubMLST.org website and their applications. Wellcome Open Res. 2018;3:124(124); doi: <https://doi.org/10.12688/wellcomeopenres.14826.1>.

12. Cody AJ, Bray JE, Jolley KA, McCarthy ND, Maiden MCJ. Core genome multilocus sequence typing scheme for stable, comparative analyses of *Campylobacter jejuni* and *C. coli* human disease isolates. J Clin Microbiol. 2017;55(7):2086-97; doi: <https://doi.org/10.1128/JCM.00080-17>.
